# Plastic flow and the skyrmion Hall effect

**DOI:** 10.1038/s41467-020-14587-4

**Published:** 2020-02-06

**Authors:** C. Reichhardt, C. J. O. Reichhardt

**Affiliations:** Theoretical Division and Center for Nonlinear Studies, Los Alamos National Laboratory, Los Alamos, NM 87545 USA

**Keywords:** Nanoscience and technology, Nanoscale devices, Magnetic devices

## Abstract

Skyrmions in chiral magnets are a particle-like texture that has been attracting growing interest due to their novel dynamics and possible applications. Here, we discuss the role of disorder and skyrmion-skyrmion interaction in governing their motion under an external drive.

Skyrmions are particle-like textures originally proposed by Tony Skyrme to produce baryons and mesons in a high energy physics field theory^1^. Much of the same underlying physics can arise in condensed matter, resulting in the appearance of skyrmions in a range of different systems. The most studied example is skyrmions in chiral magnets^2–4^. Due to their particle-like nature, high mobility, small size, and existence at room temperature in certain materials, magnetic skyrmions hold great promise for a variety of applications as information carriers or in logic devices^4,5^. Skyrmions have unique dynamical properties similar to those found in spinning objects, including a strong Magnus force which causes the skyrmions to develop velocity components that are perpendicular to an applied driving force. This produces a skyrmion Hall angle analogous to the Hall angle found for electrons in a magnetic field^4^. The mechanical analog of this effect is observable in a thrown spinning ball, which follows a trajectory that curves away from the throwing direction; however, relatively little is known about the dynamics of a collection of interacting particles with Magnus-dominated dynamics in the presence of a disordered landscape^6,7^. The skyrmion Magnus force also has implications for applications such as race track memories^5^, since the Magnus-induced motion must be compensated in order to control skyrmion transport in such devices.

## Drive dependent skyrmion Hall angle

In 2015, Muller et al.^8^ and Reichhardt et al.^9^ numerically studied moving skyrmions interacting with defects or pinning, and found that the quenched disorder induces a side jump effect or spiraling motion of the skyrmions, producing a drive dependence in the skyrmion Hall angle. At small drives, the skyrmion Hall angle is nearly zero, but it increases with increasing skyrmion velocity, and as shown in refs. ^9–11^, at high drives it reaches a plateau at the theoretical disorder-free value. These same workers found that collections of interacting skyrmions can exhibit plastic flow with coexisting mobile and pinned skyrmions. Soon after, the drive dependence and high drive saturation of the skyrmion Hall angle was directly observed in experiments^12,13^. Much of the initial numerical work involved a point particle or Thiele model for the skyrmions; however, actual skyrmions have a specific finite size and can exhibit internal modes or shape distortions, which can produce a dependence of the skyrmion Hall angle on the size of the skyrmion. Smaller skyrmions are expected to have larger Hall angles^4^, and if application of a drive changes either the size or the shape of the skyrmion, the skyrmion Hall angle could vary with drive, as proposed in^13^. This mechanism differs from the pinning-based mechanism considered previously. The skyrmion Hall angle may also depend on the nature of the skyrmion flow. Certain types of ordered flow could exhibit a skyrmion size dependence, while the disorder inherent in plastic or creep flow could wash out the effect of skyrmion size.

## One sample, many skyrmion sizes

**Fig. 1:**
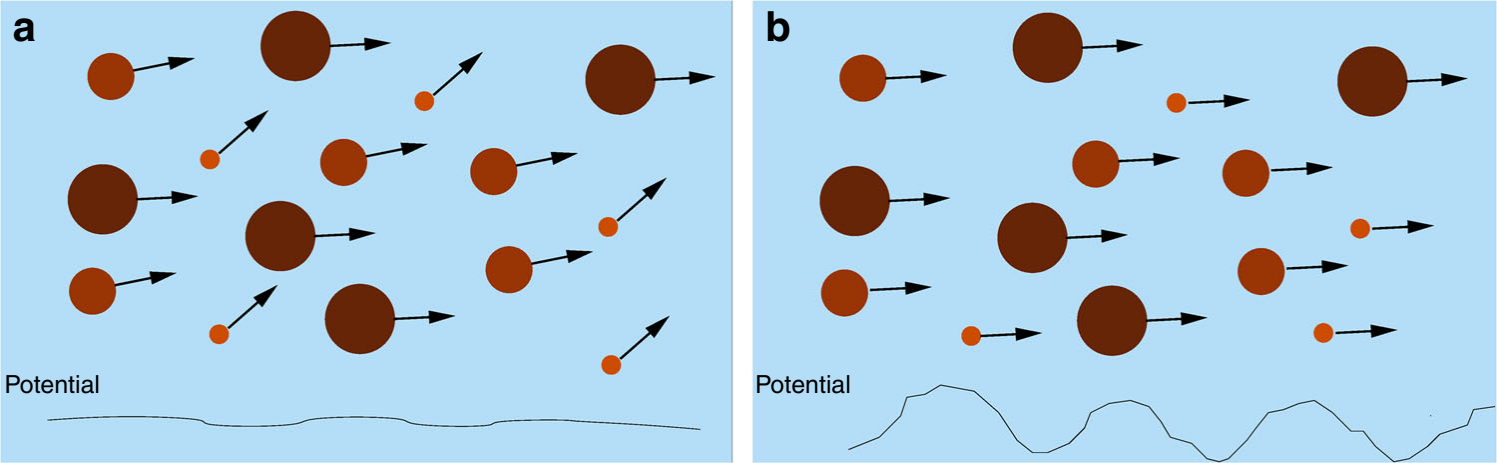
Illustration of the motion of skyrmions with different sizes in the presence of disorder. Schematic of the experiments by Zeissler et al.^14^, who use imaging to examine moving skyrmions with different diameters. The skyrmions move at an angle with respect to the drive known as the skyrmion Hall angle. **a** The results expected if the skyrmion Hall angle depends on the size of the skyrmions, with different size skyrmions moving at different angles. This can occur if the background potential is weak. **b** The observed results in which the skyrmion Hall angle is independent of skyrmion diameter. The skyrmions undergo plastic motion due to strong interaction with the pinning sites, implying a strong underlying disordered background.

In order to understand how pinning affects individual skyrmions, skyrmion–skyrmion interactions, and the evolution of the skyrmion Hall effect, Zeissler et al.^14^ examine skyrmions in multilayer systems that are prone to disorder. The key feature of this system is that it permits the coexistence of skyrmions of varied size ranging from 35 to 825 nm in diameter, making it possible to study the size dependence of the skyrmion Hall angle in simultaneous measurements of all of the sizes that are present in the sample. Figure 1a shows a schematic of the system containing skyrmions of different sizes sitting on a disordered substrate. If the skyrmion diameter determines the skyrmion Hall angle, then different skyrmion sizes will move at different angles, as illustrated in Fig. 1a. Zeissler et al. drive the skyrmions and use direct imaging to extract the skyrmion flow paths and corresponding skyrmion Hall angles. They find that although the skyrmion Hall angle increases with increasing drive, it is independent of the skyrmion diameter, as shown schematically in Fig. 1b, indicating that pinning and skyrmion–skyrmion repulsion are the dominating effects in determining the skyrmion Hall angle drive dependence. Zeissler et al. repeat their experiments under a variety of conditions and multiple drive cycles in order to obtain enough statistics to build reliable distributions and show the robustness of their results. By combining different images from the same sample, Zeissler et al. map the skyrmion trajectories and find a repeatable pattern, indicating that individual skyrmions are following easy-flow channels through the same underlying disordered background. Continuum-based simulations have also provided evidence that pinning plays a dominant role in the behavior of the skyrmion Hall angle, with a drive-independent skyrmion Hall angle appearing in the absence of quenched disorder, and strong drive dependence appearing in strongly pinned samples^11,15^.

## Probing the importance of pinning

These results indicate that the interaction of mobile skyrmions with pinning is the dominant factor controlling the drive dependence of the skyrmion Hall angle; however, there are still many questions that need to be addressed. For example, it is not known what is the characteristic length scale of the disorder in the landscape. One possibility is that if this length scale is associated with pinning by the granularity of the films, and if this is larger than the size of the largest skyrmion, then the size of the skyrmion may not matter; however, in other systems with a much smaller disorder length scale, skyrmions with different diameters could exhibit different dynamics. It is also possible that the skyrmion size dependence emerges only at much higher skyrmion velocities than those studied in ref. ^14^, due to the washing out of the pinning effectiveness at high drives similar to what is found for vortices in type-II superconductors^7^. To address these issues, future experiments could be performed with artificially created pinning sites of well-controlled size and geometry, where the skyrmion Hall effect can be studied under a systematic change of the pinning length scale. Other types of imaging or transport measurements could be used to study skyrmions in a high velocity regime where strong thermal effects are important which could thermally smear out the effectiveness of the pinning. The work in ref. ^14^ also suggests that although the skyrmion motion in the plastic flow regime is disordered, it is also repeatable due to the static pinning landscape, suggesting that the fluctuations or noise associated with the skyrmion motion could have specific repeatable signatures determined by the underlying disorder which could be of use for skyrmion memory devices.

## Device applications and beyond

Along with other measurements of the skyrmion Hall angle drive dependence, the results in ref. ^14^ have implications for interpreting electrical transport data, since a changing skyrmion Hall angle would need to be taken into account when trying to determine the changes in the topological Hall effect as a function of drive^4,6^. This work could also lead to new ideas on how to create tailored pinning arrays that guide skyrmions or even on how to control the skyrmion Hall effect in order to enhance the performance of certain devices. Other questions that can be addressed include the difference between dense interacting skyrmion lattices compared to isolated skyrmions, whether there are specific scaling relations for the evolution of the skyrmion Hall angle, and whether there is a thermal creep regime that is distinct from a plastic flow regime. It is also interesting to ask whether application of a cyclic drive leads to completely reversible skyrmion motion or whether there is an onset to chaotic dynamics as observed in other driven many-body systems^16^. If a system can be identified in which the skyrmion Hall angle is dependent on skyrmion size, then a pinning landscape could be introduced that would allow the sorting of skyrmions by size in order to create topological filters. Other systems besides skyrmions exhibit Hall effects under a drive, such as quantum crystals, charged particles, and vortices in superconductors and superfluids, and it would be interesting to explore whether a drive dependence of the Hall angle also occurs in these systems.
